# Scanning electron microscope-based evaluation of eggshell quality

**DOI:** 10.1016/j.psj.2024.103428

**Published:** 2024-01-05

**Authors:** Prem Lal Mahato, Tina Weatherby, Kristen Ewell, Rajesh Jha, Birendra Mishra

**Affiliations:** ⁎ Department of Human Nutrition Food and Animal Sciences, College of Tropical, Agriculture and Human Resources, University of Hawaii at Manoa, Honolulu, HI, USA; † Biological Electron Microscope Facility, Pacific Biosciences Research Center, University of Hawaii at Manoa, Honolulu, HI, USA

**Keywords:** eggshell-quality, mammillary, mineral, palisade, scanning electron microscope

## Abstract

The eggshell is the outermost covering of an egg that provides physical and chemical protection. It is a major source of calcium and minerals for the growing embryo during incubation. The egg industry suffers from a considerable economic loss due to poor eggshell quality. Therefore, developing an accurate and precise method of determining eggshell quality is crucial in improving eggshells in subsequent generations of breeding stock. Hence, this study aimed to develop a method to accurately and precisely determine 1) eggshell thickness using a scanning electron microscope (**SEM**) and 2) eggshell mineral components using an SEM-Energy Dispersive Spectrometry (**EDS**) system. Four types of table eggs (*N* = 48; 12 eggs/group): Cage-Free Organic from the US Mainland (**CFO-M**) and Hawaii (**CFO-H**), Caged Non-Organic from the US Mainland (**CNO-M**), and Hawaii (**CNO-H**) were sourced from the grocery store. Approximately 0.5 mm^2^ pieces of eggshells from the equator region of the egg were taken and processed for visualization under the SEM. Three distinct layers of eggshell were identified under SEM: the outermost cuticle, the middle palisade, and the innermost mammillary region. The results showed that CFO-H eggs have a greater eggshell thickness (380.43 ± 2.69 µm) and effective thickness (306.28 ± 4.15 µm). Similarly, the mammillary knob count was denser in CNO-H eggs (186 ± 23.02 knobs/0.5 mm^2^). Calcium (97.36 ± 0.17%) was the highest among minerals in lower palisades (**LP**). The magnesium concentration was lowest in the LP region, whereas the phosphorus concentration was highest in the upper palisades. Our study established a scientific method to assess the eggshell quality and biochemical characteristics of eggs through SEM and EDS. This method can be used as a marker for selecting superior parent stock to improve eggshell quality in subsequent generations of breeding stock.

## INTRODUCTION

Eggshell formation involves intricate communication among reproductive hormones, nutrition, and environment (Mishra et al., 2019). After ovulation of the yolk from the ovary, the egg formation occurs in the oviduct. The oviduct consists of the infundibulum (site of fertilization), magnum (synthesis and deposition of albumen), isthmus (shell membrane deposition), and shell gland/uterus (eggshell biomineralization) (Yu and Marquardt, 1973; Gautron et al., 1997). After 5 to 6 h of ovulation, the newly forming egg absorbs water from the isthmus and uterine fluid, creating close contact with the surrounding tissues. Eggshell formation starts in the uterine lumen and takes about 19 to 20 h to complete. For eggshell biomineralization, calcite crystals are deposited in and around the outer eggshell membrane and are regulated by OC-116, OTOP2, CALCB, STC2, and ATP2C2 genes (Sah et al., 2018). It is completed in 3 phases, namely, the initial phase (formation of the mammillary region), the rapid growth phase (formation of part of the palisade region), and the terminal phase (completion of palisade and cuticle deposition) (Gautron et al., 2021).

An eggshell is approximately 95% calcium carbonate, 3.5% organic compounds, and the remaining 1.5% are other minerals and nonmineral elements (Damaziak and Marzec, 2022). An eggshell is the first line of protection against external forces and microbial invasions. In addition to the shell membrane, it holds inner contents such as albumen and yolk. The strength of an eggshell is incredibly important for both table and hatchery eggs. The quality and integrity of an egg rely heavily on the strength of its shell for handling, storage, and transport. It is a well-established fact that eggshell quality is significantly affected by various factors such as age, breed, feed, and housing type. In addition to these factors, the duration of egg storage has also been found to have a crucial impact on eggshell quality (Wengerska et al., 2023). It has been estimated that more than 10% of eggs are lost due to poor eggshell quality, causing huge economic losses (Ahmad and Balander, 2003). Apart from providing protection, the eggshell is vital in providing essential minerals to the developing embryo. The growing embryo absorbs the calcium and other microminerals from the innermost mammillary layer of eggshells. Hence, the mineral composition of an eggshell has a dominant role in the bone and hard tissue development during the incubation period. Moreover, a supplement of minerals and vitamins through feed has been found to increase eggshell strength and thickness significantly (Swiatkiewicz and Koreleski, 2008; Stefanello et al., 2014; Xiao et al., 2014). This emphasizes the importance of this fragile yet crucial structure.

Evaluating eggshell quality is crucial, but assessing it accurately and precisely is challenging. Traditionally, thickness and strength measurements have been used to determine eggshell quality. However, a nondestructive (dynamic stiffness) method involves striking an egg to create resonance and detect cracks (Bain, 2005). Quasi-static compression tests and eggshell failure mechanisms have been commonly used for over 50 yr and continue in laboratory-based studies. The method compresses the egg between 2 flat platens at a constant speed. The maximum load before its macroscopic crack is recorded as the breaking strength of an eggshell (Voisey and MacDonald, 1978). Moreover, the utilization of nondestructive ultrasonic technology has demonstrated superior measurement repeatability. It is highly recommended that this method be utilized precisely at the point that is located 45° from the broader end of the egg (Kibala et al., 2015). The mineral composition of eggshells exhibits variations that are influenced by the color of the shell. This is due to the fact that the pigmentation of the shell is directly linked to the mineral content (Drabik et al., 2021). The thickness of an eggshell is a combination of 3 distinct regions, and the strength is the result of various mineral compositions and thicknesses. Therefore, an advanced method is necessary to ensure the need for measuring individual regions (**UP**—upper palisade, **LP**—lower palisade, and **M**—mammillary region) of an eggshell and to determine the accurate and precise eggshell quality. Hence, the study aimed to develop a method to measure the ultramicroscopic structure and determine the mineral concentration in different regions of the eggshell.

## MATERIALS AND METHODS

### Eggshell Preparation for Scanning Electron Microscopic (SEM) Analysis

Four varieties of A-grade eggs were randomly sourced from a local grocery store (*n* = 12/egg types). These include a) Cage-free-Organic from the Mainland (**CFO-M**); b) Cage-free-Organic from Hawaii (**CFO-H**); c) Caged, Non-Organic from the Mainland (**CNO-M**); and d) Caged-Non-Organic from Hawaii (**CNO-H**). The eggs were broken in the broad end to discard the albumen and yolk. The inner surface of the eggshell was washed at least 3 times with distilled water. The eggshell membrane was gently peeled off using forceps. The equator part of the eggshell was divided into 3 pieces (5 mm × 5 mm straight-edge piece). These pieces were boiled in 2% sodium hydroxide for 10 min to remove the remaining shell membranes. The shells were washed using distilled water and then kept in a box containing desiccator overnight for drying. The dried eggshells were then mounted vertically (the straight edge on the top) on an aluminum stub using conductive carbon paste. The mounting was done so that the raster rotation function of the SEM would allow the eggshell's inner, outer, and thickness layers to be seen. Raster rotation allows user to rotate the sample on the stage at various angles to maintain a constant perpendicular viewing angle for uniform visualization. The dried and mounted shells were coated with gold/palladium in a Hummer 6.2 sputter coater for 45 s, and the shells were visualized under a cold cathode Field Emission SEM (Hitachi S-4800, Tokyo, Japan) at an accelerating voltage of 5 kV.

### Measurement of Thickness and Knob Density

The outer surface of the eggshell was taken as a reference, making it face up and aligned parallelly with the screen's upper boundary. Two types of instruments were used to measure the overall thickness: an SEM scale and an ABS Digmatic Caliper (Mitutoyo, Tokyo, Japan). The parameters for measuring the ultrastructure of eggshells are defined in Table 1. The palisade region extends from the mammillary region to the beginning of the cuticle region. The cuticle layer was integrated into the palisade layer during effective thickness measurement. Mammillary layer was measured form the innermost point of eggshell to the point where mammillary fissure ends. For the width of a mammillary knob, measurement was taken from the beginning of the fissure between 2 knobs, perpendicularly extending to the adjacent fissure. Before capturing the images of the inner surface (knobs), the angle was adjusted to make it perpendicular to the electron beam. The mounted shells were of different heights, so adjustments were needed before their visualization. The SEM image of the inner surface was taken and imported in for knob numbers, and the count function in Infinity Analysis Luminous 6.5.2 (© 2007-2016 Lumenera Corporation, Ottawa, ON, Canada) was used with its manual counting tool.

**Table 1:** The ultrastructure of eggshells and their measurement.

Eggshell ultrastructure	Measurements
Cuticle	The outermost layer of an eggshell is not measured separately
Palisade	Extends from the endpoint of the cuticle region up to the beginning of the mammillary region
Effective layer	Extends from the outermost region (cuticle) to the beginning of the mammillary region (Figure 2)
Mammillary thickness	Extends from the end of the mammillary fissure to the innermost region of the shell where it attaches with the shell membrane (Figure 3)
Mammillary width	It is the horizontal distance between 2 adjacent mammillary fissures at the end points (Figure 3)
Overall thickness	It includes effective thickness and mammillary thickness (Figure 2)
Knob	Innermost pointed ends that make attachment with shell membrane (Figure 4)
Normal knob	Knobs that are rough, and extends to the effective layer
B type knobs	Round and mostly smooth knob that sticks at the innermost region. They do not extend toward the effective layer

To determine the overall eggshell thickness, mammillary thickness, and mammillary width, measurements were taken at 30/30 spots. Additionally, we counted 3 spots per egg for both normal and B-type knobs to ensure precise and accurate data. The effective layer was not measured; however, we calculated it by subtracting the average mammillary thickness from the overall average thickness of the shell.

### Scanning Electron Microscope-Energy Dispersive Spectrometry System and Mineral Measurements

The mineral composition of the eggshell was determined by using an SEM with Energy Dispersive X-ray Spectroscopy (**SEM-EDS**) system using an Oxford Ultim Max 170 EDS system mounted on the Hitachi S-4800 SEM, Tokyo, Japan. The elements to be measured were predefined, and the coated elements were excluded from the detection system.

Three distinct parts were selected in the thickness of the eggshell to measure mineral composition. The selection areas were variable, but the calculation was in weight percentage, representing the percentage of elements in the selected area. Preselected elements were divided into macrominerals (sodium, magnesium, phosphorus, sulfur, potassium, and calcium), microminerals (manganese, iron, copper, and zinc), and nonmineral elements (aluminum, silicon, vanadium, nickel).

### Technical Adjustment

To ensure accurate and precise measurements of eggshell thickness, specific parameters were used. A consistent 5 kV accelerating voltage setting was used while adjusting the working distance and magnification for the overall thickness, effective thickness, and mammillary thickness. Fixed parameters like a 30 mm working distance and 150× magnification have produced accurate results by exposing an accurate 0.5 mm^2^ area for the knob counts. SEM-EDS analysis was performed at a 15 mm focal distance (recommended by the system) and 20 kV power, using the “Point and ID” function in Oxford Instrument Aztec microanalysis software (Oxford AZtecLive version 5.1). The power required for this system was determined by the formula “2.5 times kα,” where kα is the element-associated value of the highest mass element to be measured. For this study, zinc was the highest mass element, having a kα value of 8, so the power required was 2.5 × 8 = 20 kV.

### Statistical Analysis

The data collected were analyzed using 1-way ANOVA in R-Studio version 4.3.0. The normality of the data distribution was checked using the Shapiro-Wilk test. For non-normally distributed data, the Kruskal-Wallis test was used followed by the Dunn test with Bonferroni corrections. A significance level of p ≤ 0.05 was considered to indicate a significant difference in all tests performed.

## RESULTS

### Overall Thickness of an Eggshell

**Figure 1:**
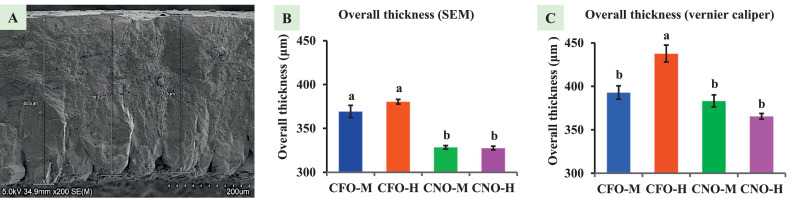
Overall eggshell thickness measured by 2 methods. (A) Representative image of SEM picture of an entire eggshell thickness; Graph showing overall thickness of eggshells measured by (B) SEM, (C) Vernier caliper. Different superscript represents significant difference (*P* < 0.05). SEM = scanning electron microscope; CFO-M = Cage-Free Organic from Mainland; CFO-H = Cage-Free Organic from Hawaii; CNO-M = Caged Non-Organic from Mainland; and CNO-H = Caged Non-Organic from Hawaii.

Eggshell thickness affects overall quality, with thicker shells indicating better eggs. Using SEM, eggshells were visualized as 3 regions, as shown in Figure 1A. SEM-based measurement of eggshell thickness showed that CFO-M (369.27 ± 6.97 μm) and CFO-H (380.43 ± 2.69 μm) were significantly thicker (*P* < 0.01) than that of CNO-M (328.47 ± 1.92 μm) and CNO-H (327.57 ± 2.10), as shown in Figure 1B.

The vernier caliper was used to measure the overall eggshell thickness, and its accuracy was compared with the SEM-based data. The overall eggshell thickness measured by the vernier caliper was significantly thicker (*P* < 0.01) in CFO-H (437.78 ± 9.80 μm) eggs as compared to CFO-M (392.96 ± 7.73 μm), CNO-M (383.33 ± 6.96 μm), and CNO-H (365.56 ± 3.30 μm) as shown in Figure 1C.

### Palisade Thickness and Effective Thickness

**Figure 2:**
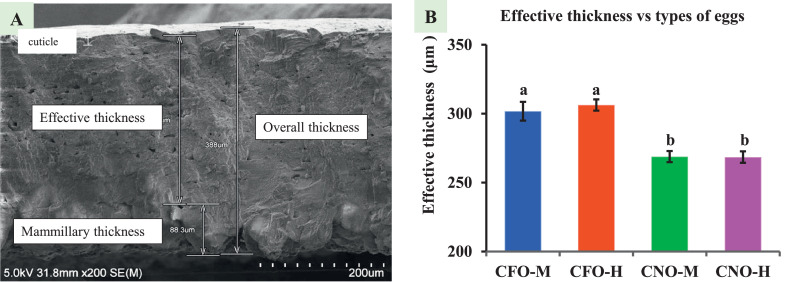
Effective eggshell thickness. (A) SEM image of the thickness of an eggshell showing effective thickness (290), mammillary thickness (83.3), and overall thickness (308); (B) Graph showing effective layer thickness. Different superscript represents significant difference (*P* < 0.05). SEM = scanning electron microscope; CFO-M = Cage-Free Organic from Mainland; CFO-H = Cage-Free Organic from Hawaii; CNO-M = Caged Non-Organic from Mainland; and CNO-H = Caged Non-Organic from Hawaii.

The effective thickness collectively includes the palisade and cuticle regions of the shell (Figure 2A). The effective thickness of CFO-M (301.75 ± 6.78 μm) and CFO-H (306.28 ± 4.15 μm) are significantly higher (*P* < 0.01) than that of CNO-M (268.83 ± 4.01 μm) and CNO-H (268.47 ± 4.16 μm) as shown in Figure 2B. Effective thickness occupied 80 to 82% of overall regions throughout the thickness, while the mammillary layer covered the rest.

### Mammillary Thickness

**Figure 3:**
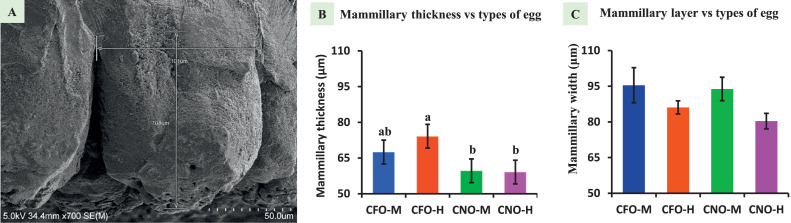
Mammillary knob measurement. (A) SEM image of an eggshell knob, showing knob thickness (vertical line) and knob width (horizontal line); Bar graph showing (B) mammillary thickness, (C) mammillary width. Different superscript represents significant difference (*P* < 0.05). SEM = scanning electron microscope; CFO-M = Cage-Free Organic from Mainland; CFO-H = Cage-Free Organic from Hawaii; CNO-M = Caged Non-Organic from Mainland; and CNO-H = Caged Non-Organic from Hawaii.

The mammillary layer is the innermost layer that attaches to the shell membrane and provides minerals to the growing embryo. Figure 3B explains that among different types of eggs, CFO-H (74.15 ± 3.25 μm) possesses a substantial mammillary layer thickness (*P* = 0.005) in comparison to CNO-M (59.63 ± 3.60) and CNO-H (59.1 ± 3.08 μm) eggs, while CFO-M (67.52 ± 3.53 μm) mammillary thickness is not significantly different from any type of eggshells. Unlike the overall thickness, the mammillary layer is impossible to measure using the traditional method.

**Figure 4:**
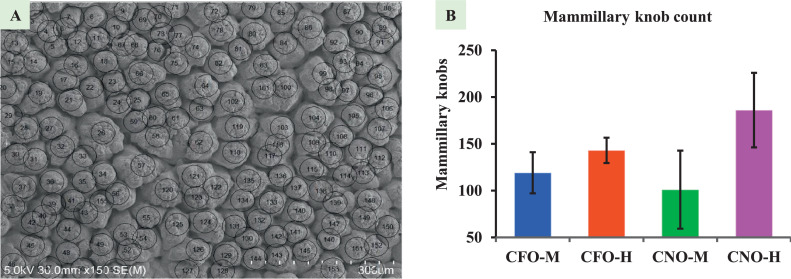
Mammillary knob count analysis. (A) SEM image of the inner surface of an eggshell showing knobs (encircled); (B) Graph showing mammillary knob counts. Different superscript represents significant difference (*P* < 0.05). SEM = Scanning Electron Microscope; CFO-M = Cage-Free Organic from Mainland; CFO-H = Cage-Free Organic from Hawaii; CNO-M = Caged Non-Organic from Mainland; and CNO-H = Caged Non-Organic from Hawaii.

### Mammillary Knob Width

Mammillary knob width represents the distance between 2 fissures created by the knobs, as shown in Figure 3A. The knob extends upward to the column and coincides with the column width. The widest knob noted for the CFO-M (95.43 ± 7.37 μm), followed by CNO-M (93.84 ± 4.93 μm), CFO-H (86.09 ± 2.77 μm), and CNO-H (80.33 ± 3.28 μm) (Figure 3C). None of these measurements were statistically different from each other.

### Mammillary Knob Counts

The normal mammillary knobs originate from the biomineralization initiation point (keratan sulfate). Its count can also be referred to as the knob density. The number of knobs was the highest (186.00 ± 23.02 knobs/0.5 mm^2^) in CNO-H eggs, followed by CFO-H (143.00 ± 7.81 knobs/0.5 mm^2^), CFO-M (119 ± 21.93 knobs/0.5 mm^2^), and CNO-M (101.00 ± 24.11 knobs/0.5 mm^2^) (Figure 4). Despite a variation in counts, knob counts were not significantly different among the egg types. The cone-shaped knobs that extend to the eggshell cuticle and contribute to the eggshell column are the normal type of knobs, while the round knobs that do not contribute to the eggshell column and do not extend to the cuticle are B-type knobs. The B-type knobs counted in the study were highest in CFO-H (9 knobs/ 0.5 mm^2^), followed by CFO-M (4 knobs/0.5 mm^2^), CNO-H (4 knobs/0.5 mm^2^), and CNO-M (1 knob/0.5 mm^2^).

**Figure 5:**
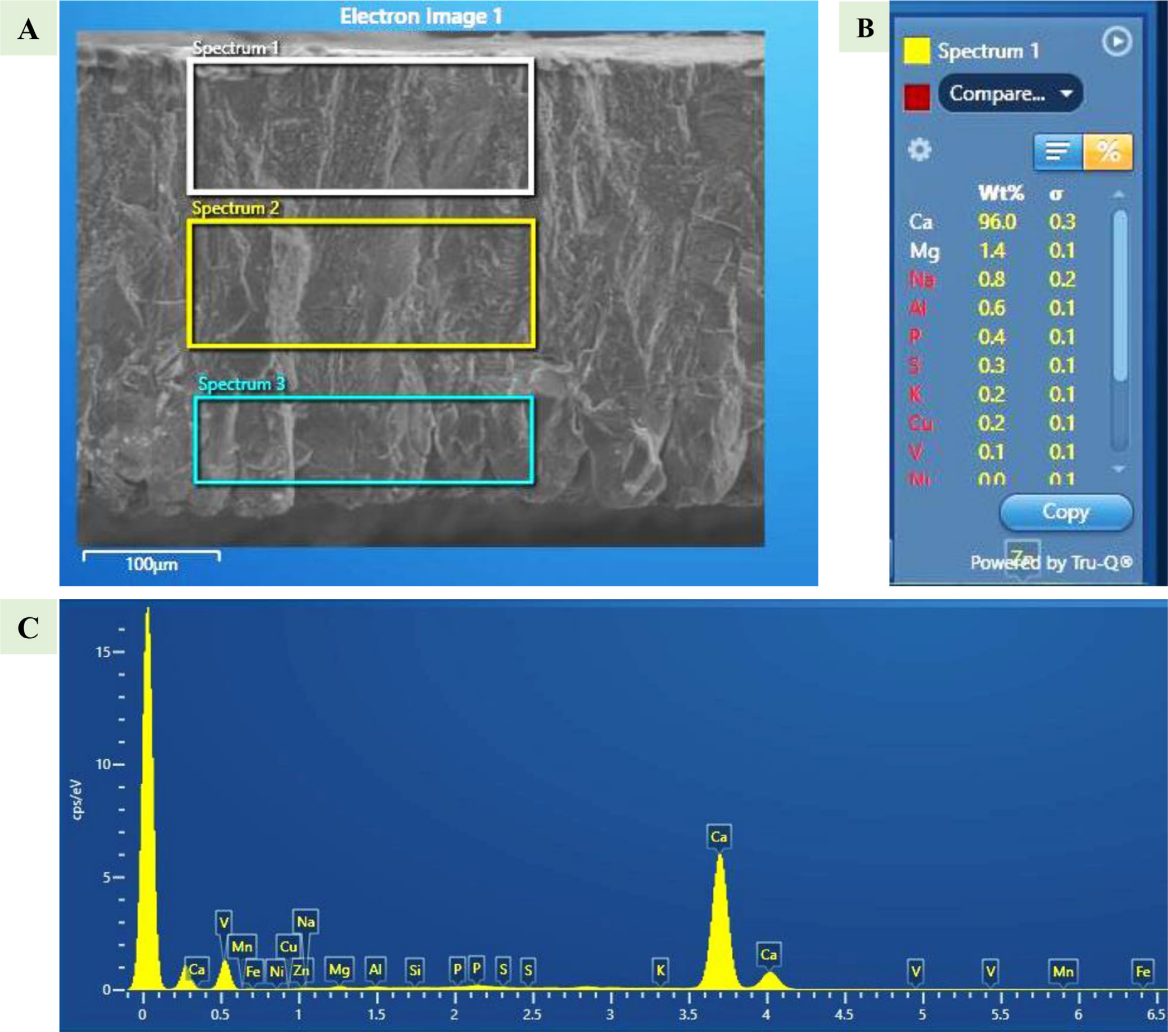
Representative picture of using SEM-EDS system for the measurement of an eggshell mineral concentration in all 3 regions. (A) Selection of (Spectrum 1) upper palisade, (Spectrum 2) lower palisade, and (Spectrum 3) mammillary region; (B) Measured values for Spectrum 1; (C) Spectrum of elements measured.

### Analysis of Eggshell Elements

**Figure 6:**
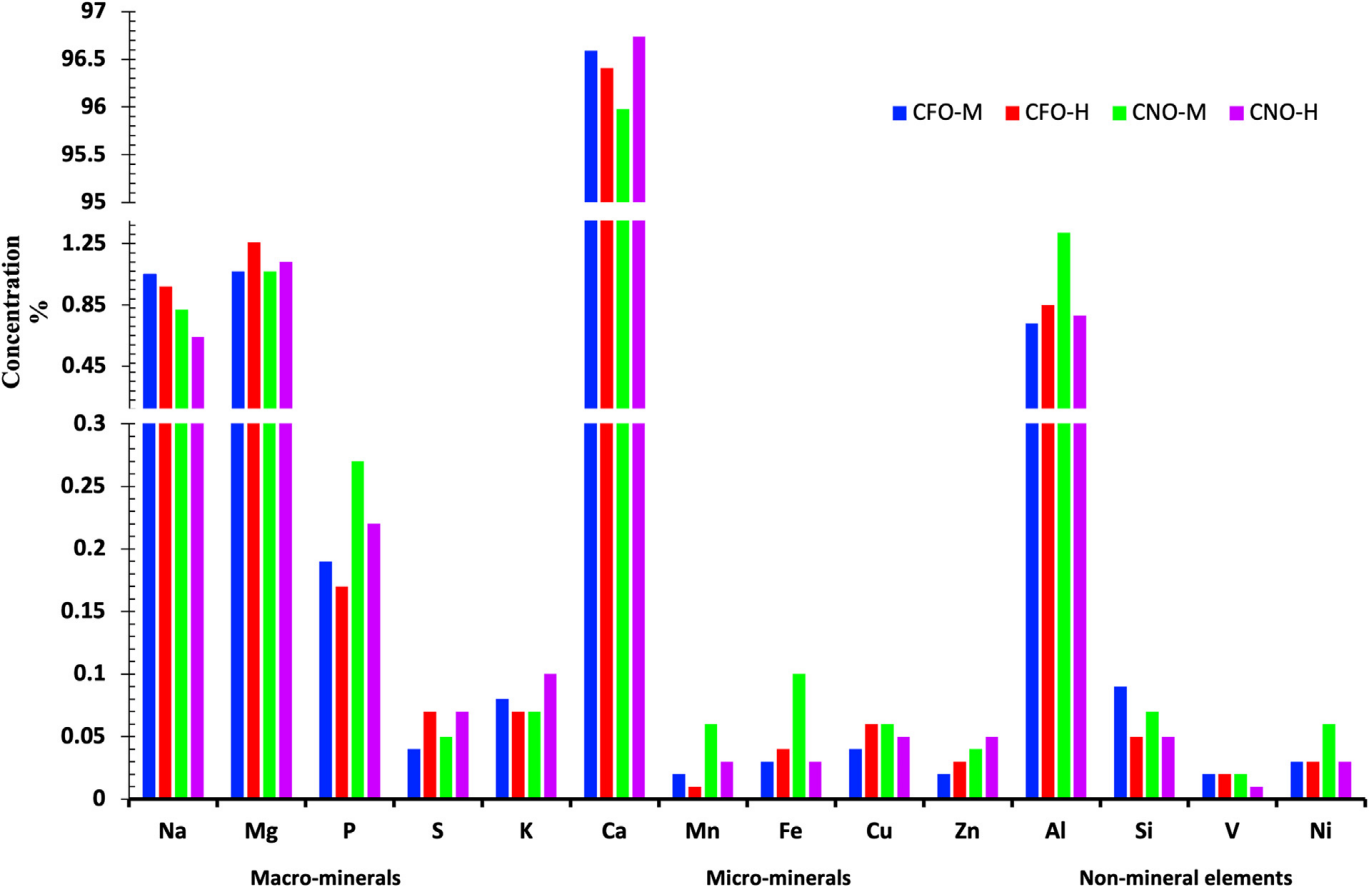
Bar graph showing the concentration of all minerals (% w/w) measured among types of eggs. Mg = magnesium; Al = aluminum; Na = sodium; P = phosphorus; Si = silicon; Cu = copper; Fe = iron; S = sulfur; Zn = zinc; K = potassium; Mn = manganese; Ni = nickel; V = vanadium; Ca = calcium; CFO-M = Cage-Free Organic from Mainland; CFO-H = Cage-Free Organic from Hawaii; CNO-M = Caged Non-Organic from Mainland; and CNO-H = Caged Non-Organic from Hawaii.

Eggshell deposition takes place in 3 phases, and mineral composition varies accordingly. Therefore, concentrations were calculated separately for 3 regions (UP, LP, and M) (Figure 5). Among all measured elements, the average concentration was the highest for calcium, followed by aluminum and magnesium. Of all selected elements, the lowest concentration was noted for vanadium (Figure 6), while some of the elements were beyond the detection level (**BDL**) in some samples. Among the macrominerals, calcium (96.33 ± 0.166%) is the highest in concentration, followed by magnesium (∼1%) and sodium (∼1%), while the remaining minerals in this group pose less than 0.3% of the concentration as shown in Table 2. The concentrations of the microminerals were less than 0.1% in all types of eggs. Among nonmineral elements, aluminum occupied the most (∼1%), while the rest were less than 0.1% of the total concentration.

**Table 2:** Macromineral concentration (W/W) in percentage among types of eggs.

Egg types	Regions	Macrominerals					
		Na	Mg	P	S	K	Ca
CFO-M	UP	1.14 ± 0.01	1.3 ± 0.17	0.34 ± 0.06	0.03 ± 0.02	0.11 ± 0.02	96.15 ± 0.27
	LP	0.94 ± 0.06	0.7 ± 0.1	0.07 ± 0.04	0.04 ± 0.02	0.07 ± 0.02	97.48 ± 0.16
	M	1.09 ± 0.05	1.37 ± 0.37	0.11 ± 0.08	0.06 ± 0.02	0.04 ± 0.01	95.58 ± 1.1
	Average	1.06 ± 0.04_x_	1.13 ± 0.16	0.18 ± 0.05	0.04 ± 0.01	0.07 ± 0.01	96.4 ± 0.43
CFO-H	UP	0.94 ± 0.11	1.4 ± 0.06	0.52 ± 0.05	0.08 ± 0.02	0.11 ± 0.01	94.58 ± 1.07
	LP	0.69 ± 0.09	0.7 ± 0.02	0.09 ± 0.03	0.04 ± 0.01	0.06 ± 0.01	97.23 ± 0.3
	M	0.83 ± 0.1	1.16 ± 0.01	0.09 ± 0.02	0.03 ± 0.01	0.02 ± 0.01	96.31 ± 0.54
	Average	0.82 ± 0.06^xy^	1.09 ± 0.1	0.23 ± 0.07	0.05 ± 0.01	0.06 ± 0.01	96.04 ± 0.53
CNO-M	UP	1.05 ± 0.25	1.5 ± 0.16	0.25 ± 0.12	0.09 ± 0.04	0.09 ± 0.01	96.14 ± 0.36
	LP	1 ± 0.26	0.92 ± 0.06	0.11 ± 0.06	0.06 ± 0.03	0.07 ± 0.01	96.97 ± 0.52
	M	0.75 ± 0.11	1.47 ± 0.14	0.11 ± 0.06	0.03 ± 0.02	0.03 ± 0	95.84 ± 0.73
	Average	0.93 ± 0.12^x^	1.3 ± 0.11	0.16 ± 0.05	0.06 ± 0.02	0.06 ± 0.01	96.32 ± 0.33
CNO-H	UP	0.77 ± 0.21	1.37 ± 0.13	0.42 ± 0.04	0.11 ± 0.04	0.16 ± 0.02	96.03 ± 0.1
	LP	0.47 ± 0.17	0.71 ± 0.03	0.08 ± 0.03	0.05 ± 0.01	0.08 ± 0.02	97.79 ± 0.23
	M	0.73 ± 0.33	1.52 ± 0.25	0.11 ± 0.01	0.05 ± 0.01	0.03 ± 0.02	95.95 ± 0.66
	Average	0.66 ± 0.13^y^	1.2 ± 0.15	0.2 ± 0.06	0.07 ± 0.02	0.09 ± 0.02	96.59 ± 0.36
Among Avg UP/LP/M (*P* value)		0.291	5.337E-07*	3.83E-08*	0.066	2.32E-07*	1.63E-04*
Among egg types Avg (*P* value)		0.00646*	0.705	0.875	0.62	0.505	0.858

Na = sodium; Mg = magnesium; P = phosphorus; S = sulfur; K = potassium; Ca = calcium; CFO-M = Cage-Free Organic from Mainland; CFO-H = Cage-Free Organic from Hawaii; CNO-M = Caged Non-Organic from Mainland; CNO-H = Caged Non-Organic from Hawaii; UP = upper palisade; LP = lower palisade; M = mammillary region; Avg = average.

Superscript * indicates a significant difference at P < 0.05, and different letters. (x, y) in superscript indicate significant differences between egg types at P < 0.05.

Throughout the thickness, CNO-H represents the highest calcium concentration, followed by CFO-M, CNO-M, and CFO-H. CFO-M and CNO-M show a significantly higher concentration for sodium (*P* = 0.0065), in comparison to CNO-H, while CFO-H is not significantly different from any type of eggshells. Tables 2 and 3 show the concentration of all elements by UP, LP, and M regions.

**Table 3:** Micromineral and nonmineral concentrations (W/W) in percentage among types of eggs.

Egg types	Regions	Microminerals				Nonmineral elements			
		Mn	Fe	Cu	Zn	Al	Si	V	Ni
CFO-M	UP	0.02 ± 0.02	0.05 ± 0.02	0.03 ± 0.03	BDL	0.71 ± 0.16	0.08 ± 0.04	0.01 ± 0	0.02 ± 0.02
	LP	0.02 ± 0	0.01 ± 0.01	0.03 ± 0.01	BDL	0.54 ± 0.01	0.03 ± 0.03	0.03 ± 0	0.02 ± 0.01
	M	0.03 ± 0	0.02 ± 0.01	0.07 ± 0.03	0.12 ± 0.04	1.19 ± 0.35	0.24 ± 0.19	0.02 ± 0.01	0.07 ± 0.02
	Average	0.02 ± 0.01	0.03 ± 0.01	0.04 ± 0.01	0.04 ± 0.02	0.81 ± 0.15	0.12 ± 0.07	0.02 ± 0	0.04 ± 0.01
CFO-H	UP	0.03 ± 0.02	0.22 ± 0.14	0.01 ± 0.01	0.05 ± 0.04	1.87 ± 1.17	0.09 ± 0.04	0.01 ± 0	0.09 ± 0.05
	LP	0.1 ± 0.03	0.01 ± 0	0.11 ± 0.03	0.02 ± 0.02	0.86 ± 0.42	0.06 ± 0.03	0.02 ± 0.01	0.04 ± 0.03
	M	0.01 ± 0	0.02 ± 0.01	0.05 ± 0.03	0.05 ± 0.02	1.14 ± 0.51	0.04 ± 0.03	0.04 ± 0.02	0.06 ± 0.03
	Average	0.04 ± 0.02	0.08 ± 0.05	0.06 ± 0.02	0.04 ± 0.01	1.29 ± 0.42	0.06 ± 0.02	0.02 ± 0.01	0.06 ± 0.02
CNO-M	UP	BDL	0.03 ± 0.01	0.05 ± 0.01	0.01 ± 0.01	0.7 ± 0.22	0.04 ± 0.03	0.02 ± 0.01	0.03 ± 0.02
	LP	0.01 ± 0.01	0.05 ± 0.02	0.07 ± 0.02	0.06 ± 0.05	0.73 ± 0.22	0.04 ± 0.03	0.02 ± 0.01	0.02 ± 0.02
	M	0.02 ± 0.01	0.03 ± 0.02	0.06 ± 0.02	0.04 ± 0.01	1.41 ± 0.56	0.08 ± 0.03	0.04 ± 0.02	0.03 ± 0.01
	Average	0.01 ± 0	0.04 ± 0.01	0.06 ± 0.01	0.04 ± 0.02	0.95 ± 0.22	0.05 ± 0.02	0.02 ± 0.01	0.03 ± 0.01
CNO-H	UP	0.05 ± 0.03	0.03 ± 0.02	0.04 ± 0.03	0.05 ± 0.02	0.68 ± 0.09	0.07 ± 0.04	0.02 ± 0.01	0.05 ± 0.01
	LP	0.02 ± 0.01	0.02 ± 0.01	0.06 ± 0.02	0.06 ± 0.04	0.62 ± 0.02	0.01 ± 0.01	0.01 ± 0.01	0.03 ± 0.03
	M	0.02 ± 0.01	0.05 ± 0.02	0.05 ± 0.01	0.03 ± 0.02	1.37 ± 0.32	0.09 ± 0.07	BDL	0.01 ± 0.01
	Average	0.03 ± 0.01	0.03 ± 0.01	0.05 ± 0.01	0.05 ± 0.01	0.89 ± 0.15	0.06 ± 0.03	0.01 ± 0	0.03 ± 0.01
Among Avg UP/LP/M (*P* value)		0.7646	0.106	0.0772	0.114	0.025*	0.09	0.5125	0.258
Among egg types Avg (*P* value)		0.329	0.854	0.706	0.906	0.899	0.945	0.321	0.233

Mn = manganese; Fe = iron; Cu = copper; Zn = zinc; Al = aluminum; Si = silicon; V = vanadium; Ni = nickel; CFO-M = Cage-Free Organic from Mainland; CFO-H = Cage-Free Organic from Hawaii; CNO-M = Caged Non-Organic from Mainland; CNO-H = Caged Non-Organic from Hawaii; UP = upper palisade; LP = lower palisade; M = mammillary region; Avg = average.

Superscript * denotes significant differences (P < 0.05) within eggshell regions (UP, LP, and M).

The variation of concentration throughout UP, LP, and M regions reveals that calcium was significantly higher in LP in comparison to M and UP (*P* = 0.0002). Moreover, the concentration of phosphorus was highest (*P* < 0.05) in UP, as compared to M and LP. Furthermore, magnesium was lowest in LP (*P* < 0.05), aluminum was highest in the M region (*P* < 0.05), and potassium level was detected to be significantly different in all 3 regions of the eggshells.

## DISCUSSION

The enhancement of eggshell quality is crucial for the sustainability of both poultry meat and egg industries. In this study, we developed accurate methods of accessing eggshell quality using SEM and SEM-EDS technology. Under SEM, an eggshell is magnified thousands of times without missing any details. SEM technique enables a thorough visualization of 3 principal regions of the eggshell, namely the cuticle, palisade, and mammillary regions. The cuticle serves as the first outermost protective layer with antimicrobial activity. The middle palisade region is the thickest region of the shell, which plays a significant role in eggshell strength. The innermost mammillary region is a cone-shaped structure that holds onto the eggshell membrane and gives strength. The cuticle of an eggshell plays a significant role in preventing microbial entry into the egg through the pores. However, its thickness is negligible and cannot be relied upon alone. Therefore, it is combined with the palisade region to obtain the effective thickness of the eggshell. The effective thickness is significantly related to the puncture force, and the favorable ultramicroscopic structure for a sound eggshell is a close association between the organic shell membrane and mammillary knob (Carnarius et al., 1996). Microporosity of the palisade region determines the density of an eggshell, and since it is the thickest layer, it consumes a significant amount of calcium. Lower density indicates a lower concentration of calcium as well. Bain (1992) concluded that the crack starts from the cone-shaped innermost mammillary region, radiating to the outermost cuticle surface. The thickness and concentration of elements in all these regions affect the eggshell quality. The study focuses on developing a methodology for egg-shell quality determination, not on finding better eggs; however, the comparison made among the egg types in this study validates the method's functionality.

### Eggshell Thickness

Eggshell thickness measured by SEM and vernier caliper showed a similar trend: higher thickness for the cage-free organic eggshell; however, the measurement range is lower for the SEM, showing less variable measurement from the SEM scale. Although there is a similar trend, there is a difference in the eggshell thickness measurements obtained through the SEM and vernier caliper methods. The observations suggest that the vernier caliper approach results in higher thickness values than those obtained using the SEM method. This implies a discrepancy between the 2 techniques. Both instruments measured the same eggshell piece, but SEM measured the edge, and the vernier caliper cannot measure the same surface. It measures the thickness by holding the upper and lower surface, while SEM measures the surface length along the thickness. The vernier caliper had a high chance of human error because of its manual setting. To accurately measure the eggshell thickness, a fixed and movable jaw must touch the shell surface. An eggshell is curved, and vernier caliper jaws are not; if we measure a piece of curved eggshell, there is a high chance of a gap between one of the jaws and the eggshell. Moreover, the brittle eggshell broke easily when we tried to adjust the jaw using the thumb screw. On the other hand, by using SEM, we can magnify the eggshell thickness and measure it with the least amount of error. Zhang et al. (2019) compared the strong and weak eggshells and found that the stronger eggshell had higher thickness. Similarly, Liao et al. (2013) concluded that the thicker eggshell better supports hatchability. Hence, determining the exact thickness of an eggshell is necessary to determine the strength and hatchability of the egg, which can be performed easily and with the least error by using SEM.

### Effective Thickness

Effective thickness comprises the palisade region and cuticle that covers more than a third of the whole thickness. A thicker and more effective layer is desired for better hatchability and strength of an eggshell (Zhang et al., 2019). The highest effective layer belongs to Cage-Free and Organic eggshells (**CFO**), while both thinner eggshells were from Caged and Non-Organic eggs (**CNO**). Because the traditional method cannot measure individual regions (UP, LP, and M) of an eggshell, change in effective thickness is only measured in the SEM method. Cage-Free and Organic birds are less stressed and get direct sunlight. When exposed to sunlight, birds get vitamin D, which in turn helps increase calcium concentration in the blood (Jing et al., 2022). Cage restricts birds’ movements and exercise, and they are not exposed directly to the sun. The presence of the vitamin D receptor in the uterine gland (Bar, 2008) makes them more vulnerable to calcium depletion.

USDA-certified organic eggs are laid by hens raised without animal products or laboratory-synthesized chemicals (Morris, 2016). The thickness of organic eggs is higher than that of nonorganic eggs due to several factors. Organic hens receive more vitamin D, which positively affects eggshell thickness. Moreover, the environment in which organic hens are raised is less stressful, and the type of feed they consume also plays a role in producing thicker eggshells. Overall, these factors contribute to the increased eggshell thickness of organic eggs.

### Mammillary Thickness

The mammillary region is crucial for embryo growth by providing microminerals and maintaining eggshell integrity. The thinner mammillary region supports the strength (Parsons, 1982), while the thicker region supports the hatchability (Liao et al., 2013). Moreover, the smaller mammillary thickness is associated with early fusion and is believed to have a higher fracture toughness (Feng et al., 2020). Thicker mammillary regions support thicker eggshells, resulting in enhanced strength. Conversely, thinner mammillary regions promote early fusion, which is desirable for eggshell durability. CFO-H eggs have the highest effective and mammillary thickness, indicating their stronger nature and better hatchability. CNO eggs have smaller mammillary regions, indicating their strong shell but compromised hatchability. For the same overall thickness, if the mammillary thickness is smaller, the effective thickness will be higher. While the studies explain separate contributions of effective and mammillary thickness, none can explain their combined or relative contribution to strength and hatchability. However, a smaller mammillary region generally supports early fusion and a bigger effective thickness. The birds with thinner eggshells might have a strategy to increase their eggshell strength by reducing the mammillary thickness and increasing the effective thickness, with a cost of lowered hatchability. Hence, the measurement of the mammillary region can reveal the strength and hatchability potential of an egg, and it can be used in the selection of a breeder, laying the most favorable egg for hatching.

### Mammillary Knob Width and Count

Sulfate glycosaminoglycan is the origin point of a mammillary knob in the outer eggshell membrane; a higher amount of this compound indicates a higher number of mammillary knobs or lowered width of the mammillary knob. Measuring the width of knobs poses a significant challenge due to their nonround shape. The knobs are often elliptical, and it is difficult to determine whether the measured surface corresponds to the narrower or broader end of the ellipse. This uncertainty results in a wide range of measurements, and no significant changes were noticed among types of eggs. However, both egg types from the Mainland had wider knobs than those from Hawaii. Zhang et al. (2017) supplemented organic and inorganic manganese and increased the eggshell-breaking strength due to decreased mammillary width, decreased mammillary layer, and increased effective layer. The study also mentioned that knob width decline was related to the increase in sulfate glycosaminoglycans on the membrane, and its positive correlation was observed with eggshell-breaking strength. The measurement of knob width in high-strength and low-strength eggshells showed a thickness of 57 μm and 83 μm, respectively (Zhang et al., 2019), which clears the role of smaller knob width in eggshell strength. Among the measured widths in this study, CNO-H has the lowest width, indicating its stronger nature. The density of knobs is determined by sulfate glycosaminoglycan density, which in turn decide how wide a knob will be, and further knob width resolves how wide a column width will be. Therefore, knowing the knob width will be very useful in finding stronger eggshell markers. Knob density, or the number of knobs in the unit area in the eggshell inner surface, can also indicate the density of keratan sulfate (sulfate glycosaminoglycans). The highest knob density observed in this study was from CNO-H, and the lowest was from CNO-M. The knob density and knob width are related because narrower knobs help fit more knobs in a small area, increasing knobs per unit area. Like knob width, both egg types from the Mainland had lower knob density than eggs from Hawaii. The regular and higher number of these knobs in the unit area of the eggshell is considered a stronger eggshell (Zhang et al., 2022). Regular and uniform distribution of keratan sulfate is necessary for a stronger eggshell (Robinson and King, 1970; Ha et al., 2007). Studies have conflicting ideas about the number of knobs in the eggshell and their strength, as explained by Simons (1971); the higher the number of knobs higher the shell strength. The higher number of knobs increases the interstitial space making higher crack initiation spots (Van Toledo et al., 1982). The knob density was 235, 178, and 144 per mm^2^ in 30, 60, and 78 wk old layer hens, respectively (Park and Sohn, 2018), indicating their decreasing trend over increasing age. The variation in knob number in this study could be due to age variation. B-type knobs are reported to be higher in number in aged birds (Park and Sohn, 2018; Zhang et al., 2022), but the effect of a specific number of B-type knobs on strength has not been reported yet. Without the SEM method, measuring mammillary thickness, knob width, and knob density is impossible; hence the method will be a milestone for improving eggshell quality.

### Elemental Analysis Using Scanning Electron Microscope-Energy Dispersive Spectrometry

In the traditional method, the eggshell minerals are measured by crushing eggshells and using specialized spectrometry (Klingensmith and Hester, 1985; Drabik et al., 2021). The mineral concentrations of individual regions were impossible to measure using traditional methods, which is very important because each region of the eggshell has its specific role in keeping an eggshell strong and hatchable. Measuring a single concentration of the entire eggshell and assuming that the same concentration is responsible for the strength or supply of minerals to the embryo will be misleading. If the eggshell is thinner, the minerals must be deposited in the smaller section. Thinner effective thickness could be one of the many causes of higher calcium concentration in CNO-H eggs. An experiment supplying oral liquid potassium chloride (0.4% KCl) to reduce heat stress showed an increased eggshell thickness (Dai et al., 2009). However, they could not show the concentration variation in the eggshell due to the supplement. Potassium concentration was higher in the UP region of CNO-H eggs, followed by CNO-M eggs; both had smaller eggshell thickness. Although oral potassium supplement positively affects eggshell thickness, their higher concentration in eggshells may have the opposite effect on thickness. Overall magnesium concentration is highest in CFO-H, which has the thickest effectiveness and overall thickness. Higher magnesium concentration in song thrush bird eggs compared to blackbird eggs has been concluded to be one of the reasons for the higher reproductive efficiency of song thrush (Damaziak and Marzec, 2022). The higher thickness and magnesium in CFO-H eggs show a higher potential for superior reproductive performance. For the average concentration of minerals, the LP region had the highest calcium concentration, as compared to the upper palisade and mammillary region. Similarly, potassium is variable, higher in UP, followed by LP and M. The role of these individual elements in certain regions of an eggshell has not been examined yet. Simkiss (1961) concludes that the mammillary layer supplies 80% of the total calcium required for the normal growth of the embryo. Similarly, it has been hypothesized that trace minerals can have a significant role in early fusion (Mabe et al., 2003). The mineral composition of the shell contributes to the eggshell coloration, especially zinc combines with other compounds to color the eggshell covering (Drabik et al., 2021). Despite the positive effect of manganese on the knob characteristics (Zhang et al., 2017, 2018), the highest knob number was noted in CNO-M, which has the second-highest manganese concentration in this study. Zinc is one of the significant components of the carbonic anhydrase enzyme (**CA**), which helps in forming bicarbonate, and is a necessary component for calcium carbonate. Furthermore, the extra magnesium supplied in feed has shown better eggshell quality in aged hens, but the ultramicroscopic change because of that specific supplement has not been documented yet (Kim et al., 2013). Each mineral has its specific role in eggshell strength, hatchability, and thickness. Through this methodology, future studies might find some element markers as an indicator of eggshell strength.

## CONCLUSIONS

We used both conventional (vernier caliper) and advanced (SEM) methods to measure eggshell thickness, with the latter showing only minor variation in measured values. Only the SEM method successfully measured all of the underlying regions (UP, LP, and M), and we used the EDS system aiding in the SEM to measure the mineral concentration of the individual regions. This combination has proven to be an effective approach for in-depth analysis of mineral samples. Using the SEM method, we can find the correlation between various factors potentially affecting the eggshell quality. Moreover, the method will help determine the marker for more robust and thicker eggshells with better hatchability. Despite being an expensive technique, this method can improve eggshell quality in breeders and successive generations, benefitting the poultry industry. Further research is needed to understand the impact of age, breed, feed, and housing on egg quality, including mineral concentration, eggshell thickness, and hatch parameters.
